# Pain management challenges in a patient with mucopolysaccharidosis IVA

**DOI:** 10.1002/ccr3.9214

**Published:** 2024-07-28

**Authors:** Marcus Gurgius, Sarah Donoghue, Robyn A. Wallace

**Affiliations:** ^1^ College of Health and Medicine University of Tasmania Hobart Tasmania Australia; ^2^ Tasmanian Pain Clinic St Helen's Private Hospital Consulting Suites Hobart Tasmania Australia; ^3^ Metabolic Diseases Unit, General Medicine Department Royal Melbourne Hospital Parkville Victoria Australia; ^4^ Department of Biochemical Genetics Victorian Clinical Genetics Services Parkville Victoria Australia; ^5^ General Medicine Department Calvary Lenah Valley Hospital Hobart Tasmania Australia

**Keywords:** acupuncture, chronic pain, disability, pain, pain management

## Abstract

It is important to consider all potential pain management modalities including alternative treatment on managing complex pain presentations. Acupuncture is a treatment modality that may result in reduction of pain in patients with significant medical comorbidities due to MPS IVA.

## 1 INTRODUCTION

Mucopolysaccharidosis IVA (MPS IVA), also known as Morquio Syndrome, is a rare autosomal recessive lysosomal storage disease, with an estimated prevalence ranging from 1/40,000 to 1/200,000 births.^1^ MPS IVA is caused by a bilallelic variants in the GALNS gene, which encodes the galactoamine‐6‐sulfate sulfatase enzyme, leading to intracellular accumulation of the glycosaminoglycans (GAGs) keratan sulfate and chondroitin‐6‐sulfate.^1^ Intracellular accumulation of these GAGs causes progressive damage to cells and tissues by both primary and secondary destructive processes. Characteristic disease manifestations include skeletal disease, tracheal and smaller airway deposition, organomegaly, ligamentous laxity, and connective tissue deposition.^2^

Pain is a common feature in MPS IVA, but it is often inadequately evaluated and managed.^2^ There are limited data available on the pathophysiology and epidemiology of pain in children with MPS and even less among adults. Individuals with a diagnosis of MPS experience different types of pain (nociceptive, neuropathic, and mixed) in relation to the complications of the disease.^2^ In 2014, a patient‐reported outcome survey in patients with MPS IVA highlighted that joint pain was experienced by 64% of children and was inversely related to the frequency of wheelchair use.^3^

This case focuses on pain management in an adult with MPS IVA by a multidisciplinary team including specialist pain management using laser acupuncture therapy. Written informed consent was given by the patient involved to document their de‐identified case history.

## 2 CASE HISTORY/EXAMINATION

The patient is an Australian Caucasian who was referred for specialist pain management at the age of 37 years on a background of MPS VIA with significant skeletal and connective tissue changes impacting on pain management and quality of life. At the time of referral, the patient was experiencing persistent, gradually worsening widespread mechanical joint pain predominantly affecting the large joints of both lower limbs and the cervical spine region. The pain in the lower limb joints manifested after weight‐bearing activity, with the bilateral hip and knee pain limiting the patient's physical and social functioning abilities. The severity of the pain had previously resulted in presentation to the emergency department for evaluation. They continued to be independent with some functional limitations requiring mobility aids and environmental modifications at home, in their office‐based occupation, and in their car. Their functional mobility was also limited by shortness of breath due to mixed obstructive and restrictive respiratory disease from MPS IVA. They were able to independently ambulate 40 m on the 6‐min walk test, before experiencing limitation due to pain and dyspnoea. They used a motorized wheelchair for longer walking distances. An exercise physiology program was implemented by the patient to improve a walking distance from 40 to 120 m.

The patient also experienced a chronic intermittent headache approximately once per month. The headache was often throbbing in nature, incapacitating and occasionally associated with nausea and photophobia. Cervical spine region pain and menstruation were identified as the main triggers for this headache.

The diagnosis of MPS IVA was made at the age of 3.5 years following investigation for short stature with features of a skeletal dysplasia with hip dysplasia noted on hip x‐rays from 6 months of age and features of dysostosis multiplex noted at the time of evaluation by a clinical geneticist. Biochemical testing on peripheral blood leukocytes indicated markedly decreased Galactose‐6‐sulphate sulphatase 1.7 pmol/min/mg (normal range 52–140 pmol/min/mg) in the context of normal levels of β‐galactosidase and arylsulphatase‐A, providing a biochemical diagnosis of MPS IVA. Genetic testing subsequently confirmed a molecular diagnosis with two variants reported in the *GALNS* gene. The genetic variants reported in this case included a pathogenic variant c.376G>T (p. Glu126Ter) and a variant that was reported as likely pathogenic c.865A>G (p. Asn289Asp). At the time of diagnosis, conservative management was the only established treatment option, with enzyme replacement therapy and bone marrow transplantation becoming treatment options years later.

The patient had significant skeletal changes of MPS IVA with a severe gibbus deformity of the spine and short stature with a final adult height of 105 cm. They had spinal disease with C2 and C4–C7 foraminal narrowing and had previously experienced transient symptoms of paraesthesia after a rapid forward flexion movement. They also had significant respiratory comorbidities with obstructive sleep apnoea and tracheal stenosis from a goiter, asthma and mixed restrictive, and obstructive lung disease.

There had been multiple surgeries in childhood and early adolescence to manage their skeletal complications, including for dorsal decompression of the craniocervical junction and occipito‐cervical fusion with a Ransford loop at the age of 6 years, for bilateral severe genu valgum at 8–9 years and to correct ankle deformity. No further operative management was used after the age of 19 years. Mobility aids were used from the age of 10 years, initially with elbow crutches and progressing to electric wheelchair use after the age of 12 years. The skeletal surgeries did not result in reduced use of mobility aids or improved function. There were significant concerns about general anesthesia risk for further surgery due to the potential for failed intubation or extubation and the potential for spinal cord compression. Treatment with enzyme replacement was considered when it was made available through the life‐saving drugs program in Australian in 2015, but it was felt that the enzyme replacement therapy was not going to make a substantial change to the patient's quality of life.

The patient was only taking simple analgesia with oral paracetamol as required for both joint pain and headache management with limited benefit at the time of her referral to the pain specialist. The patient was mindful of the adverse effects of other analgesics and of the limitations of surgery with the significant risks of airway morbidity and potential spinal cord compression.

An initial examination showed painful and limited cervical spine range of movements. The patient had bony deformities involving her bilateral ankles, bilateral knees, and bilateral hips. There were no signs of active inflammatory arthritis. No tenderness was elicited over the greater and lesser occipital nerves bilaterally. There were no signs of sensory or motor radiculopathy.

## 3 METHODS (DIFFERENTIAL DIAGNOSIS, INVESTIGATIONS, AND TREATMENT)

Investigation with whole‐body bone scan with SPECT CT (single‐photo emission computed tomography) reported moderately increased scintigraphic uptake in bilateral hands, wrists, elbows, knees, ankles, and feet in keeping with osteoarthritis, low‐grade uptake in bilateral hip joints with collapse of the femoral heads due to acetabular dysplasia, with no scintigraphic uptake in the cervical spine.

In our case, the patient had predominantly mechanically‐driven, weight‐bearing, joint‐related nociceptive pain secondary to MPS IVA and intermittent migraine. Physical tolerance was limited by pain and/or shortness of breath. An empirical trial of laser acupuncture therapy was offered as an alternative pain management modality.

The patient was initially treated with five laser acupuncture therapy sessions over a period of 5 weeks. Three to six acupuncture points were utilized during each session. By the end of these sessions, the patient had reported a 50% reduction in baseline multi‐joint pain and headache. The therapy course was interrupted by the patient experiencing a COVID‐19 infection. With the withdrawal of treatment, the patient reported more pain and this impacted on their ability to mobilize. Laser acupuncture therapy sessions were eventually resumed and were reported to improve pain and facilitate a more rapid return to office‐based work.

The therapy sessions were initially undertaken on a weekly basis then on a fortnightly basis. Through the laser acupuncture therapy course, four acupuncture points appeared to be the most beneficial. These four points continued to be utilized over the period of 8 months.

## 4 CONCLUSION AND RESULTS (OUTCOME AND FOLLOW‐UP)

The Brief pain inventory (Table 1) and McGill pain questionnaire (Table 2) were utilized before and after the laser acupuncture therapy.

**TABLE 1:** Brief pain inventory.

Brief pain Inventory	Before Acupuncture Therapy	After Acupuncture Therapy
Pain location	Generalized joint pain particularly affecting ankles, hips, knees, low back, shoulders, arms, and neck	Pain became more localized to the ankles and hips, with total clearance of pain in other areas
Pain scoring in the last week (0 = no pain, 10 = worse pain imaginable)	8/10	3/10
General activity scoring in the last week 0 = does not interfere 10 = completely interferes	7/10	3/10
Sleep scoring in the last week	4/10	0/10
Enjoyment of life scoring in the last week	5/10	0/10
Mood scoring in the last week	7/10	2/10

**TABLE 2:** McGill pain qsuestionnaire.

McGill pain questionnaire	Before acupuncture therapy	After acupuncture therapy
What does your pain feel like?	Dull‐aching pain	Dull (rather discomfort) pain
How does your pain change with time?	Constant pain, but exacerbated by movements, walking, and cold weather	Pain became periodic/intermittent, not exacerbated by movements and walking, but can still be exacerbated by cold weather
How strong is your pain?	Distressing pain right now, at its worst and at its least.	Mild pain right now and at its least. Pain is discomforting at its worst

The patient continued to show persistence in pain reduction and there were no reported adverse effects through the entire therapy course. Current therapy sessions are performed every 3 weeks.

## 5 DISCUSSION

Musculoskeletal problems are prominent features in the mucopolysaccharidoses with many factors contributing to the development of pain in MPS IVA. Understanding the underlying pathophysiology of the condition, the limitations of operative and enzyme replacement therapy, the importance of exercise and optimizing multimodal analgesia are likely to all be important to optimizing pain and quality of life in patients with MPS IVA. From the time of diagnosis, patients with this condition require a multidisciplinary biopsychosocial approach to their physical health care, including pain management.^10^, ^11^

The evolution of the skeletal changes in MPS IVA are due the accumulation of keratan sulfate and chondroitin‐6‐sulfate, occurring predominantly in cartilage, but also present in other connective tissues.^4^ The intracellular accumulation of these GAGs leads to a destructive inflammatory process similar to inflammatory arthritis which are driven by an inflammatory cytokine response triggering apoptosis from the deposition of GAGs in cartilage and bone.^5^ This impacts on the quality and quantity of the cartilage with impaired chondrocyte function and failure of endochondral ossification.^5^ These processes impact on the growth of bone in MPS IVA, which leads to the development of short stature and significant bony changes impacting upon the entire skeleton. These same inflammatory responses could also trigger perineuronal microglial activation, which has been described as a prominent component of MPS disease and could conceivably contribute to pain.^2^

Enzyme replacement therapy (ERT) treatment, such as elosulfase alfa, has been established in some of the mucopolysaccharidoses to have some impact on improvement of bone quality and cartilage, particularly if started early in the disease process.^6^, ^7^

Treatment of the complications of MPS IVA are usually directed at managing symptoms and need to be carefully balanced to minimize harm from airway complications or spinal cord compression.^8^ Surgical interventions are common in the first two decades of life to manage skeletal complications with the neck, hips, and ankles being the most frequent sites for surgery.^8^ Up to 75% of patients with MPS diseases may need emergency or elective surgical interventions to manage complications of their condition.^1^ The use of exercise programs in MPS is an area that requires further evaluation, with some reports of improvements in movement with physical exercise programs in MPS IVA.^9^ Treatment of metabolic diseases with complementary medicines and therapies may be considered, however some patients may feel reluctant to disclose or discuss these therapies with their treating physician.^12^

There are limitations for self‐reported questionnaires to measure outcomes. Self‐report studies are inherently biased by the person's feelings at the time they filled out the questionnaire. If a person feels bad at the time they fill out the questionnaire, for example, their answers will be more negative. If the person feels good at the time, then the answers will be more positive. Another limitation is the potential for biases or inaccuracies in the data, which can occur at the source of the data and during processing.

Our patient had complex disease with airway and spinal cord involvement. Consideration of corrective surgeries, enzyme treatment, pharmacological, and non‐pharmacological treatment of pain are shaped by the influence of comorbid syndromic conditions and functional outcomes. In this case, further surgery was limited by operative risk, enzyme replacement was considered but was not pursued as there would be limited functional gains and quality of life in advanced disease, and simple analgesics were ineffective. Opioid analgesics could be associated with drowsiness, fatigue, impaired cognition, impaired functioning, addiction, tolerance and impact on established respiratory disease.

The need for a low‐risk treatment in a complex patient prompted the consideration of acupuncture as a treatment modality. Acupuncture is the stimulation of specific acupuncture points in the body. Analgesia in acupuncture is from modulation of central pain descending pathways involving endogenous opioids, serotonin, and norepinephrine.^13^, ^14^, ^15^ The efficacy of acupuncture in the treatment of different pain conditions such as migraine, abdominal pain and fibromyalgia has been reported.^15^, ^16^, ^17^ The WHO Consultation on acupuncture published a review of 225 clinical trials in 2002, concluding that acupuncture was effective for 28 diseases and beneficial for 63 others.^20^

Laser acupuncture (LA) is the use of nonthermal, low‐intensity laser irradiation to stimulate acupuncture points. It has become more common among acupuncture practitioners in recent years. LA is promoted as a safer pain‐free alternative to traditional acupuncture, with minimal adverse effects and greater versatility. LA has many features that make it an attractive option as a treatment modality, including minimal sensation; short duration of treatment; and minimal risks of infection, trauma, and bleeding complications. Future studies with high‐quality methodologies, ample sample sizes, and consistent and reproducible laser parameters are critically needed to increase understanding and establish potential future clinical applications.^19^

Despite wide use in clinical practice, acupuncture remains a controversial treatment for chronic pain. Acupuncture is effective for the treatment of chronic musculoskeletal pain, headache, and osteoarthritis pain.^15^ The therapeutic effect of acupuncture persists over time and it cannot be solely explained in terms of placebo effect.^15^ The National Institute for Health and Care Excellence (NICE) has recommended acupuncture for chronic primary pain management in its 2021 Guidelines.^18^

Although laser acupuncture was utilized as a stand‐ alone treatment in this case, it can often be used as part of multidisciplinary approach. The use of acupuncture here helped avoid opioid‐induced respiratory depression and other adverse effects, facilitating continuation of professional employment and functional independence. It also avoided the use of non‐steroidal anti‐inflammatory drugs and the potential risks of their long‐term use. Future research in this area may be needed. Referral for a course of acupuncture treatment is a reasonable option for a patient with chronic pain due to MPS IVA.^15^

## AUTHOR CONTRIBUTIONS

**Marcus Gurgius:** Validation; writing – original draft; writing – review and editing. **Robyn A. Wallace:** Conceptualization; methodology; supervision. **Sarah Donoghue:** Data curation; formal analysis; resources; writing – original draft.

## FUNDING INFORMATION

No funding was received to assist with the preparation of this manuscript.

## CONFLICT OF INTEREST STATEMENT

The authors have no relevant financial or non‐financial interests to disclose.

## ETHICS STATEMENT

Not applicable.

## CONSENT

Written informed consent was obtained from the patient to publish this report in accordance with the journal's patient consent policy.

## Data Availability

Data sharing is not applicable to this article as no new data were created or analyzed in this study.
